# Automated analysis of activity, sleep, and rhythmic behaviour in various animal species with the Rtivity software

**DOI:** 10.1038/s41598-022-08195-z

**Published:** 2022-03-09

**Authors:** Rui F. O. Silva, Brígida R. Pinho, Nuno M. Monteiro, Miguel M. Santos, Jorge M. A. Oliveira

**Affiliations:** 1 UCIBIO-REQUIMTE-Applied Molecular Biosciences Unit, Department of Drug Sciences, Pharmacology Lab, Faculty of Pharmacy, University of Porto, Porto, Portugal; 2 Associate Laboratory i4HB-Institute for Health and Bioeconomy, Department of Drug Sciences, Pharmacology Lab, Faculty of Pharmacy, University of Porto, Porto, Portugal; 3 CIBIO/InBIO, Research Centre in Biodiversity and Genetic Resources, University of Porto, Porto, Portugal; 4 Department of Biology, Faculty of Sciences, University of Porto, Porto, Portugal; 5 CIMAR/CIIMAR-Interdisciplinary Centre of Marine and Environmental Research, Group of Endocrine Disruptors and Emerging Contaminants, University of Porto, Porto, Portugal; 6 Departamento de Ciências do Medicamento, Laboratório de Farmacologia, Faculdade de Farmácia, Universidade do Porto, Rua de Jorge Viterbo Ferreira, 228, 4050-313 Porto, Portugal

**Keywords:** Software, Behavioural methods, Animal behaviour, Circadian rhythms and sleep, Pharmacology, Toxicology

## Abstract

Behavioural studies provide insights into normal and disrupted biological mechanisms. In many research areas, a growing spectrum of animal models—particularly small organisms—is used for high-throughput studies with infrared-based activity monitors, generating counts per time data. The freely available software to analyse such data, however, are primarily optimized for drosophila and circadian analysis. Researchers investigating other species or non-circadian behaviour would thus benefit from a more versatile software. Here we report the development of a free and open-source software—Rtivity—allowing customisation of species-specific parameters, and offering a versatile analysis of behavioural patterns, biological rhythms, stimulus responses, and survival. Rtivity is based on the R language and uses Shiny and the recently developed Rethomics package for a user-friendly graphical interface without requiring coding skills. Rtivity automatically assesses survival, computes various activity, sleep, and rhythmicity parameters, and performs fractal analysis of activity fluctuations. Rtivity generates multiple informative graphs, and exports structured data for efficient interoperability with common statistical software. In summary, Rtivity facilitates and enhances the versatility of the behavioural analysis of diverse animal species (e.g. drosophila, zebrafish, daphnia, ants). It is thus suitable for a broad range of researchers from multidisciplinary fields such as ecology, neurobiology, toxicology, and pharmacology.

## Introduction

The study of animal behaviour provides insights on normal and disrupted biological mechanisms. Neurological diseases, exposure to chemicals (e.g. neuroactive compounds or contaminants), environmental stressors (i.e., temperature, pH, hypoxia), and changes in light conditions may translate into behavioural alterations, which can be studied in different animal models^1–4^. Animal activity is usually monitored by video recordings or by infrared (IR)-based activity monitors. While video recordings usually require large data storages and image processing steps before data analysis^5^, IR-based activity monitors supply low-storage numerical data that can be directly analysed, thus being particularly useful for prolonged studies, detecting activity in either light or dark stages^6–9^.

The Drosophila Activity Monitor (DAM) and the Locomotor Activity Monitor (LAM) are IR-based activity monitors developed by Trikinetics (TriKinetics, Waltham, MA), which automatically count the number of IR beam crossings by small organisms. DAM and LAM can monitor animal activity for long periods of time, even in the absence of light. They have been successfully used to study the activity of different small organisms, such as flies^10^, bees and wasps^11^, ants^6^, spiders^7^, or even aquatic organisms, such as *Daphnia magna* and *Eurydice pulchra*^9,12^, and the small vertebrate *Danio rerio* (zebrafish; present study).

Most freely-available software for the analysis of the data from IR-based activity monitors focus on the evaluation of specific behaviour parameters such as circadian activity^13,14^ or sleep^15^. A recently developed software, called ShinyR-DAM, specifically created to analyse drosophila locomotor activity, greatly decreased the complexity of the analysis of data generated by the DAM, thereby making the simultaneous analysis of activity, sleep and circadian parameters accessible to novice users^16^. Since most of these software, including ShinyR-DAM, were optimized to study activity, sleep and circadian rhythmicity in drosophila, there is still a need for more versatile software. Namely, one that allows analyses customised to the behavioural specificities of distinct animal species, while also covering more diverse aspects of biological rhythmicity and transient behaviours.

In this work, we developed Rtivity, a freely available open-source software that allows a versatile and automated analysis of behavioural data obtained from IR-based activity monitors, or other sources (inc. manual or automated counting) as long as they are in a counts per time format. Rtivity allows: (i) the customization of behavioural parameters to different species, and survival analysis; (ii) the analysis of diverse biological rhythms and transient responses; (iii) the integrated analysis of multiple experiments, while also structuring data export to optimize software interoperability. Rtivity can be used either online or offline, exhibits a user-friendly interface and presents a high graphical versatility, creating publication-ready graphs.

In summary, Rtivity facilitates and enhances the versatility of behavioural data analysis, being customisable to a diverse array of animal species and experimental protocols (e.g. different light-cycles), thus being suitable to a broad range of investigators working in multidisciplinary fields of research.

## The Rtivity software

The Rtivity (R + acTIVITY) software is based on the R programming language^17^, using Shiny and the recently published Rethomics packages^18,19^. We primarily developed Rtivity to analyse the data exported by LAM and DAM, and it accepts their standard 42 column text files, called *monitor files*. However, Rtivity can also analyse other data as long as they are first converted into the monitor files format, as we detail in the user guide (see “Software availability and user guide”).

**Figure 1:**
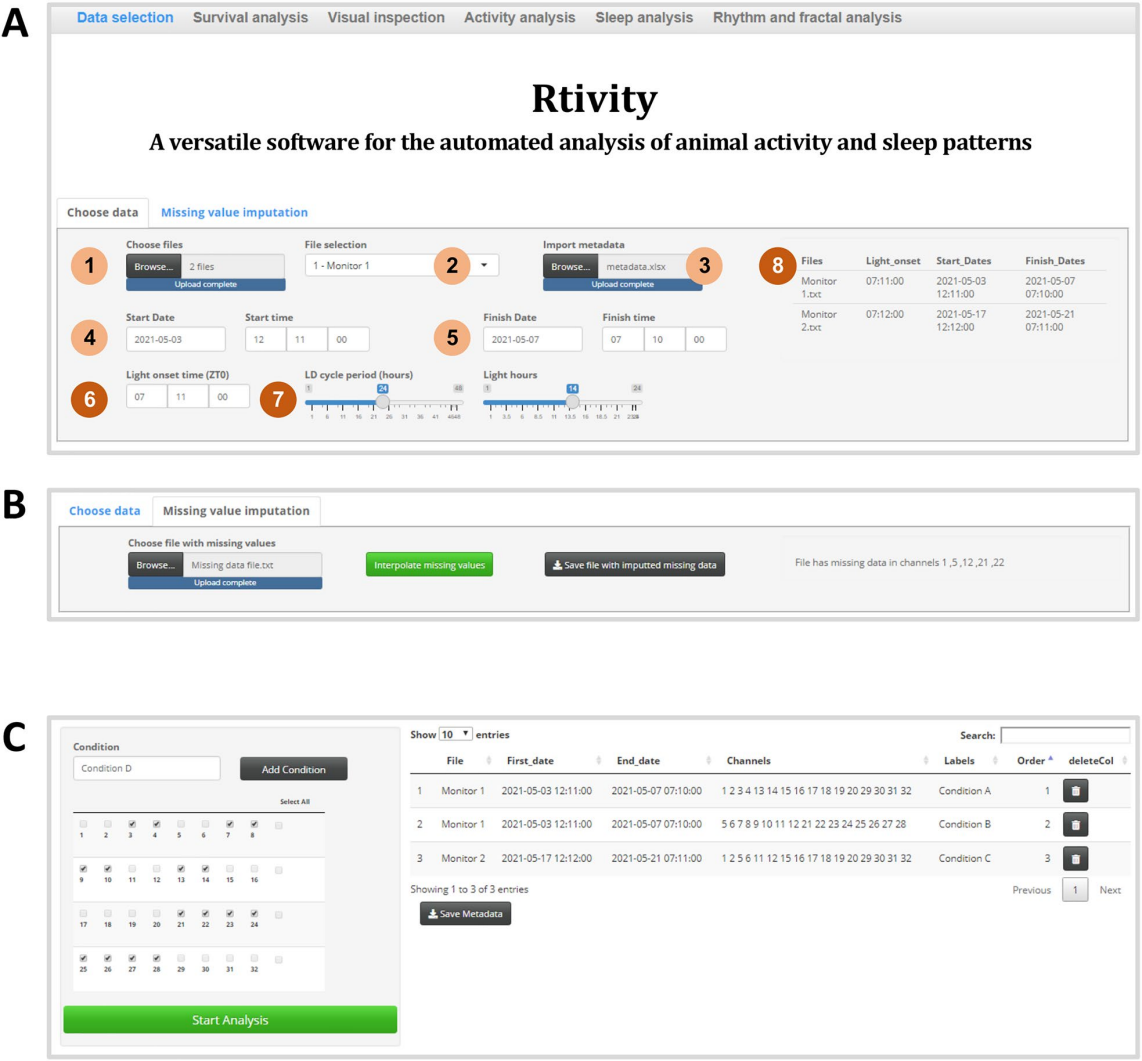
Rtivity interface. (**A)** Selection of files and analysis parameters: (1) file import; (2) file selection; (3) import metadata; (4) start date; (5) finish date; (6) light-onset time (zeitgeber time zero; ZT0); (7) light–dark (LD) cycle period and light hours per cycle; (8) display of files selected for analysis and their corresponding ZT0. (**B)** Missing values imputation. Users can upload the file, check the status message for missing values, select the option to interpolate, and save as a new monitor file. (**C)** Channel selection and grouping into experimental conditions: (left) each of the 32 channels of a LAM or DAM system can be freely selected into a user-labelled condition; (right) display of the metadata for each condition (original filename, dates, channels, condition labels, and order of conditions in the graphs), which will be imported to the Rethomics libraries for downstream analysis. Images in Figure contain screenshots of the Rtivity software developed in this study (https://ruisilva.shinyapps.io/rtivity/), assembled and illustrated by co-authors RS and JMAO using version 2.8.22 of the free image editor GIMP (https://www.gimp.org).

Rtivity presents a user-friendly interface (Fig. 1), evaluates parameters related with activity, sleep, survival, and biological rhythms, performs customisable time-continuous and column graphical representations and calculates statistics from the represented data (Table 1). In the following sections, we detail the major advantages of Rtivity, namely: (1) Data pre-processing to check file format and missing values, allowing their imputation; (2) the customization of activity and sleep parameters to different species, and survival analysis; (3) the analysis of diverse biological rhythms and transient responses; and (4) the integrated analysis of multiple experiments and software interoperability. Lastly, we explain how researchers can freely access the open-source Rtivity software, demonstration video, and user guide.

**Table 1:** Behavioural parameters, graphs, and statistics automatically computed by Rtivity.

Behavioural parameters	
**Activity**	Sum of IR-beam crossings (counts) per time-window (bin size)
Cumulative activity	Cumulative sum of IR-beam crossings since the beginning of the analysis
Activity bout	A time interval of activity occurring between 2 consecutive movement stops (stops are time intervals with zero activity that exceed a user-defined inactivity threshold, in minutes) Rtivity computes the mean activity and mean duration, in minutes, of all bouts in a specified time interval (e.g. per day, custom)
Activity period	Period (in hours) corresponding to the highest peak (power) of an activity periodogram
Activity phase	Temporal difference between the lights-on time and the activity peak time (acrophase)
Inter-daily stability	The variance of the average 24-h pattern of activity divided by the total variance
Intra-daily variability	The sum of the variance between consecutive hours divided by the total variance
Relative amplitude	The difference between the most active 10 h period (M10) and the least active 5 h period (L5), divided by the sum of M10 and L5
Scaling exponent	Slope of the correlation between the amplitude of activity fluctuations and the time-scales
**Sleep**	Time intervals of prolonged inactivity (zero IR-beam crossings) that exceed a user-defined threshold in minutes
Sleep ratio	Proportion of sleep in a specified time interval (e.g. per day, custom)
Sleep bout duration	Duration, in minutes, of sleep bouts (time interval of uninterrupted sleep)
Sleep latency	Time interval, in minutes, since lights are turned off until the start of a sleep bout
Total sleep time	Number of hours that animals were considered asleep during darkness
Wake after sleep onset	Number of hours that animals were awake during darkness, after the first sleep bout
Time of death	Last timepoint (day and time hh:mm:ss) with non-zero activity, before an animal is considered dead (time of inactivity above the death threshold)

Graphs	
Kaplan Meier	Rtivity plots survival % (y axis) over time (x axis) using the Kaplan–Meier representation
Chronograms	Rtivity chronograms are line graphs representing activity or sleep ratio (y axis) over time (x axis). Rtivity chronograms can represent the entire experimental period (Full chronogram) or only one light–dark cycle (Average LD cycle chronogram), where each time point is the average of the same time point in all cycles. The Average LD cycle chronogram is only computed for the activity parameter
Single or double plot actograms	Rtivity actograms use ribbons with a color gradient to represent activity over time (x axis) In “Survival analysis”, single plot actograms display individual channels as separate ribbons along the x axis (full time of experiment). In “Visual inspection”, the single plot actograms display the mean activity for each condition (grouping several channels). Alternatively, Rtivity can produce double plot actograms, where data for each condition is plotted twice, in a staggered manner, allowing a vertical comparison of activity patterns over time
Cumulative activity	Representation of the cumulative activity (y axis) over time (x axis)
Periodograms	Representations of the similarity (i.e. power; y axis) between data segmented in different periods (x axis). Rtivity computes the Chi-Square and Lomb-Scargle periodograms
Column graphs	Representations of a behavioural parameter (y axis) per condition, organized for example by day or by light and dark phase (x axis). Column graphs can either be bar-plots, box-plots, dot-plots (with mean and error) or simply the mean and error bars

Statistics	
The statistics associated with each graph (e.g., n, mean, median, quartiles, SD, SEM) are displayed in a table and can be downloaded as XLSX files	

### Data pre-processing

First, Rtivity checks if the uploaded files have the required format (*.txt monitor file). Rtivity reports an error when files contain missing values (empty or non-numeric data) in the columns representing activity channels. In LAM and DAM data, missing values or noise are rare (given the continuous monitoring in a closed system^20^) but may potentially occur due to IR sensor malfunction. However, Rtivity can also analyse data from other sources where noise or missing values are more common (e.g. actigraphy with wearable sensors, which can be temporarily removed or misplaced)^21^. Thus, common data pre-processing steps involve data filtering (to minimize the noise influence over the analysis^22^) and missing value imputation (to avoid wasting valuable experiments due to minor missing data^21,23^). If required, data filtering can be performed prior to uploading data into Rtivity (a commonly used method is the Kalman filter^22^, which is available in R packages^24^). Missing value imputation can be done by Rtivity, which creates a new monitor file containing interpolated values (Fig. 1B). These are computed by the *Copy Mean* method, which was shown to be robust for different types of missing data^25^.

### Customization of behavioural parameters to different species

IR-based activity monitors generate data from which researchers can evaluate animal activity patterns. However, inactivity-related parameters can also be useful to evaluate normal or disrupted animal behaviour. The method used to monitor activity largely influences both activity- and inactivity-related parameters^26^. Since animal species can markedly differ in their activity levels^6,7,10^, it is advantageous to have the software capability to adjust the time threshold used to consider an animal inactive (i.e. time without IR-beam crossings). Animal inactivity can be transient (stops), temporary (e.g. sleep) or definitive (e.g. death), thus, Rtivity allows the user selection of three different inactivity-related thresholds:

#### Stops

**Figure 2:**
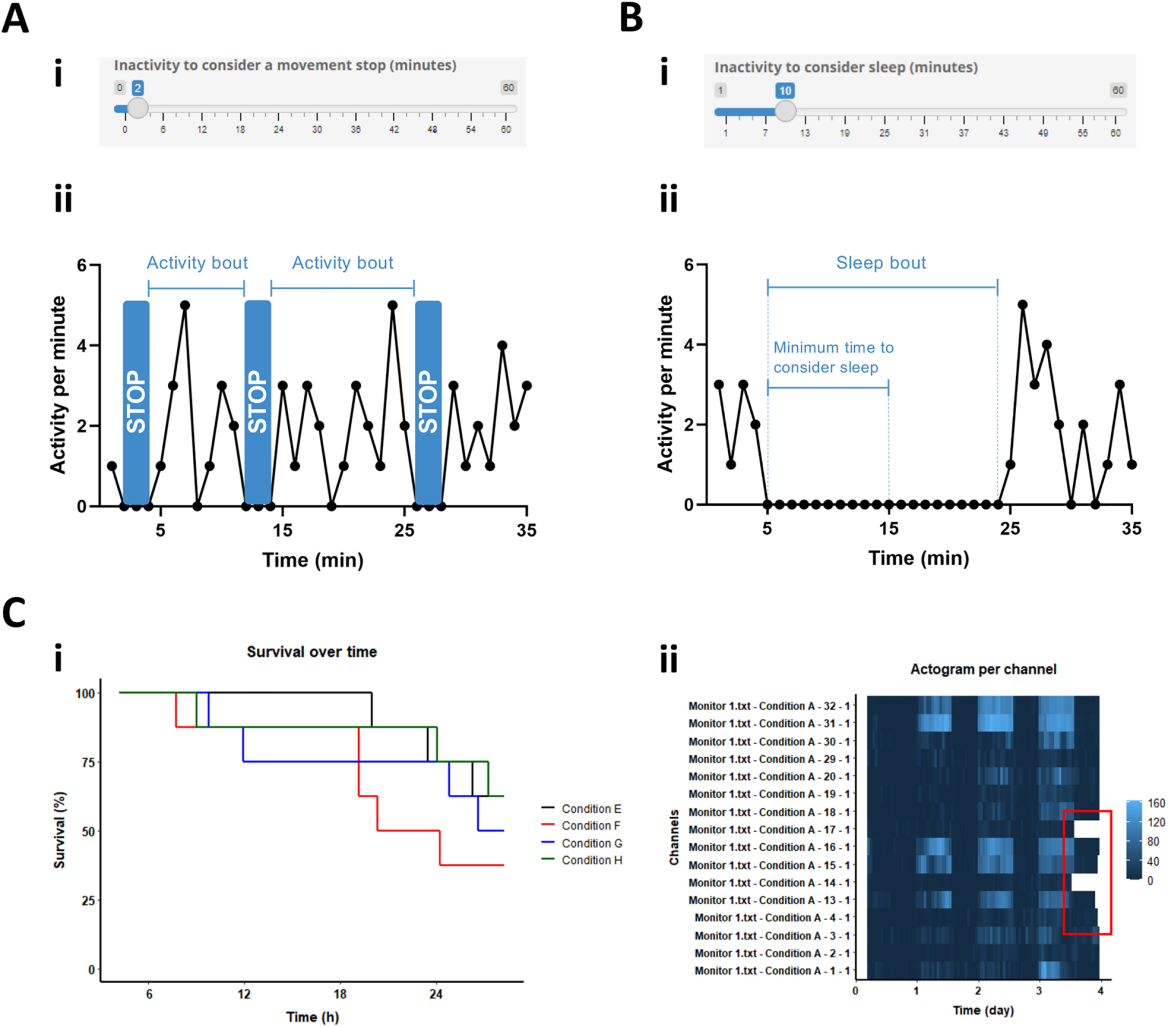
Inactivity parameters. (**A) **Movement stops and activity bouts: (i) user-defined inactivity threshold to consider a movement stop; ii) representation of activity bouts between two movement stops. (**B)** Sleep and sleep bouts: (i) user-defined inactivity threshold to consider that an animal is asleep; ii) representation of a sleep bout between two timepoints with non-zero activity. (**C)** Survival analysis: (i) survival percentage over time (Kaplan–Meier plot); (ii) representative actogram per channel, where the last inactivity periods are excluded from the representation (white sections inside the red rectangle). Data are from individual zebrafish larvae; time 0 equals 4 days post fertilization (dpf). Images in Figure contain screenshots of the Rtivity software developed in this study (https://ruisilva.shinyapps.io/rtivity/), assembled and illustrated by co-authors RS and JMAO using version 2.8.22 of the free image editor GIMP (https://www.gimp.org).

Animals with similar mean activity levels may differ substantially in the number of stops or duration of continuous movement. In Rtivity, the user can select the minimum period of time without detected activity to define movement stops (Fig. 2Ai). Rtivity uses the analysis of stops to extend the evaluation of activity to uninterrupted movement intervals—activity bouts (Fig. 2Aii)—calculating their mean activity and mean duration (Table 1).

#### Sleep

Detailed characterization of sleep can be performed in humans, rats, and mice^27,28^ using complex data (e.g. brain waves) or simpler data (e.g. activity and heart beat) analysed with mathematical models^29,30^. In small organisms, animal sleep can be generally characterized as a prolonged quiescent state, whose duration varies across species^31^. Since Rtivity was primarily developed for small organisms, we used the prolonged quiescent state definition to identify sleep^31^*.* Previous software defined 5 min as the minimum period of time to consider that an animal is asleep^15,16^. Although this definition suits drosophila^32^, different species have diverse sleep behaviours^31^ (e.g. zebrafish larvae: 10 min sleep threshold^33^). Hence, Rtivity allows users to adjust the minimum inactivity period required to consider that an animal is asleep (Fig. 2Bi). This allows species-specific evaluation of sleep-related parameters: sleep ratio, duration of sleep bouts (uninterrupted periods of sleep; Fig. 2Bii), sleep latency, total sleep time, and wake after sleep onset (Table 1).

#### Death (and sustained paralysis or experimental anomalies)

Animal inactivity can be definitive when animals become unable to move or die. In research areas such as toxicology death is viewed as an experimental result, since it may be a consequence of the exposure to exogenous compounds or to disruptive conditions. Recently developed software significantly improved the speed and efficiency of detection of dead animals by introducing a daily locomotor activity threshold, thereby allowing the automatic exclusion of dead animal data^16^. In some experiments, however, researchers may be particularly interested in the time of death, or in the behavioural changes occurring prior to death (e.g. study of time-dependent effects of a toxic compound). To accommodate such particular interests, we used the following rationale: if an animal dies at any time point during the experiment, it will have zero activity counts until the end of the experiment. Rtivity users can define a specific inactivity time (*death threshold*) to consider death for their species of interest. Rtivity will then perform a backward analysis starting at the last experimental time point (from the end of the experiment going backwards), identifying animals with a continuous inactivity period that is larger than the user defined death threshold (‘dead animals’). Next, users can choose to remove the dead animals entirely from the analysis or, alternatively, choose to remove only their inactivity data after death (still being able to assess behavioural changes occurring prior to death). Another useful feature of Rtivity is that it facilitates survival studies, by registering the survival status at the end of the experiment, and automatically computing the time of death (Table 1). This allows the graphical representation of the survival percentage over time (Kaplan–Meier plot; Fig. 2Ci). Also, Rtivity allows users to visually inspect the data by generating actograms **(**Table 1) that display the individual channels from the monitor files (Fig. 2Cii). These actograms assist users with the definition of the death threshold, and the quality control search for experimental anomalies (e.g. empty channel, IR sensor malfunction, aberrant activity data).

In summary, to customize the behavioural analysis to different species, Rtivity allows users to specify different inactivity thresholds, thus optimizing the analysis of movement, sleep and death. Rtivity also eases the analyses of diverse biological rhythms, as described below.

### Analysis of diverse biological rhythms and transient responses

The study of biological rhythms often requires continuous analysis of activity over prolonged periods, which may span several days and nights. IR-based activity monitors are particularly useful for such prolonged studies due to their ability to detect activity independently of light conditions. Moreover, they provide numerical data that can be directly analysed, thus saving time and data storage when compared with prolonged video recordings^6,9,11^. The available software used to analyse data from IR-based activity monitors, however, are often optimized to a particular model species (e.g. drosophila) and thus allow only fixed 12 h light 12 h dark (12L:12D) cycles^16^. If the researchers require the use of different light–dark cycles (e.g. 16L:8D for house fly or Daphnia^10,12^; 14L:10D for zebrafish, present study), or wish to mimic seasonal variations in photoperiod, they would benefit from a software allowing a more flexible input of light–dark cycles. Rtivity has the advantageous feature of allowing the customization of the period and light hours of each light–dark cycle (Fig. 1A). This customization is also useful for experiments where short light–dark cycles (e.g. 30 min L : 30 min D) are used as a stimulus to evoke behavioural responses^34,35^.

**Figure 3:**
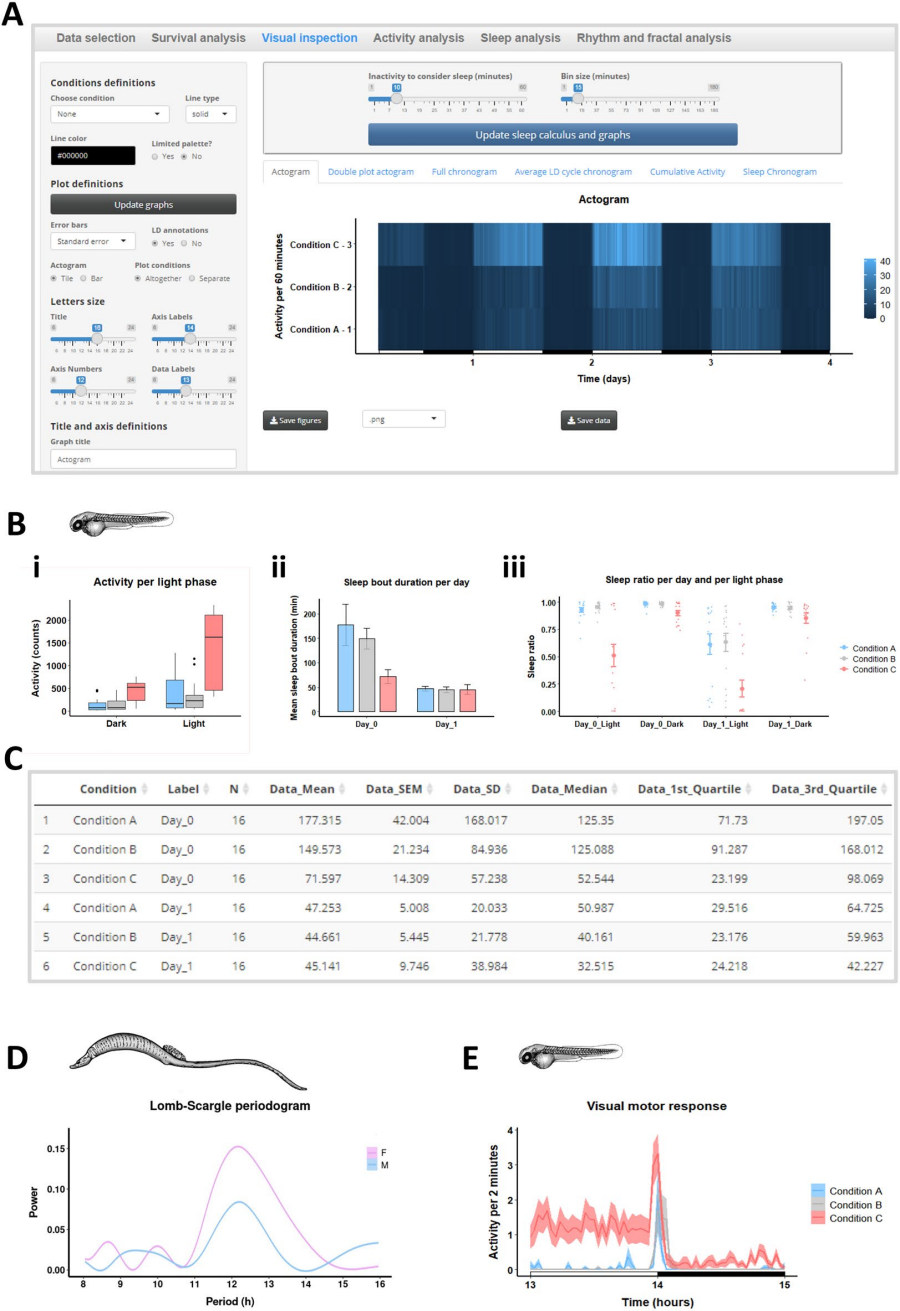
Versatility of data visualization and analysis. (**A)** Visual inspection: partial display of user inputs to customise publication-ready graphs. The actogram represents activity per 15 min with light and dark annotations (x axis). (**B)** Representation of activity per light phase (i), sleep bout duration per day (ii), and sleep ratio per day and light phase (iii); in box-plots (i), bar-plots (ii), and dot-plots (iii). Data are from individual zebrafish larvae (time 0 equals 4 dpf). (**C)** Descriptive statistics of the sleep bout duration per day (Bii). For each condition, the table includes the number of animals, mean, errors (SEM; SD), median, and other quartiles (Q1, Q3). (**D)** Lomb-Scargle periodogram of intertidal pipefish activity (adult *Nerophis lumbriciformis*), showing circatidal rhythmicity (12.4 h) for females and males (F, M). (**E)** Representative chronogram showing ‘visual motor responses’ evoked by sudden light-to-dark-transition in zebrafish (4 dpf). Zebrafish data were obtained with LAM10 (Trikinetics). Pipefish data were obtained with video recordings and illustrate the capability of Rtivity to use counts per time data from any source, if they are first converted into the standard monitor file format. Images in Figure contain screenshots of the Rtivity software developed in this study (https://ruisilva.shinyapps.io/rtivity/), and drawings by co-author NM of pipefish and zebrafish, assembled and illustrated by co-authors RS, NM, and JMAO using version 2.8.22 of the free image editor GIMP (https://www.gimp.org).

The analysis of time-continuous data usually starts by the visual inspection of graphical representations^36^. Rtivity allows a versatile exploratory data analysis, displaying time-continuous (e.g. actograms, chronograms or cumulative activity) or column graphs (e.g. bar-plots, box-plots, dot-plots, mean & error bars), which users can adjust to create publication ready graphs, as detailed in the user guide (See “Integrated analysis of multiple experiments and software interoperability”). Briefly, users may adjust axis, labels, title, condition color and linetype, and represent different error bars (Fig. 3). Time-continuous graphs can display the data acquisition time (the 0 value is the first point analysed) or the zeitgeber time (the 0 value is the first light onset time).

Biological rhythms are usually characterized by their period, rhythmic strength and phase^37^. Rtivity computes and plots the *activity period* and estimates the *rhythmic strength* with the Chi-Square or Lomb-Scargle periodograms (Table 1)^36,37^. Rtivity calculates the *activity phase* (Table 1) via the difference between the lights-on time and the activity peak time (acrophase)^38^. For this calculation, Rtivity uses the recently developed *ActCr* package^39^ to model the activity data into cosine curves (*Extended Cosinor Model*)^40^. Moreover, Rtivity computes other parameters that summarize activity rhythms: the *inter-daily stability* (IS; measures the day-to-day reproducibility); the *intra-daily variability* (IV; measures fragmentation); and the *relative amplitude* (RA; measures amplitude)^37,40^ (Table 1).

Animal activity fluctuations may present patterns that remain similar across several time scales (minutes to hours), meaning that they are scale-invariant—also known as fractal activity patterns. Such patterns have been studied in small organisms (e.g. drosophila^41^, zebrafish^42^, and *C. elegans*^43^) and in mammals (e.g. rodents, primates, humans), where disruptions have been associated with aging and neurological diseases^44–48^. Rtivity performs fractal analysis with the “Detrending Fluctuation Analysis” (DFA) method^49^, using the recently developed *nonlinearTseries* package^50^. Rtivity plots the correlation between the amplitude of activity fluctuations and the time scales, and computes the scaling exponent parameter (Table 1), which represents the scale-invariance and typically ranges from ~ 0.5 (white noise) to ~ 1.5 (rigid and excessively regular activity patterns)^44–47^.

To illustrate Rtivity graphical capability, Fig. 3 depicts data from our experiments with zebrafish larvae, showing: activity over time with automated light and dark annotations (Fig. 3A); mean activity in column graphs with data segmented by light phase (Fig. 3Bi), sleep bout duration with data segmented by day (Fig. 3Bii), and sleep ratio segmented by day and light phase (Fig. 3Biii). Moreover, in Fig. 3C, we show an example of the descriptive statistics computed by Rtivity, here referring to the graph of Fig. 3Bii.

In order to showcase Rtivity effectiveness in detecting less commonly explored biological rhythmicity, we revisited the description of the rhythmic activity patterns of an intertidal pipefish, *Nerophis lumbriciformis*^51^. After seamlessly importing the data (acquired, at the time, from video recordings) and adjusting the light–dark cycle to that experienced by the pipefish at the time of collection (13.5L:10.5D), we were able to promptly detect, in both sexes (Fig. 3D), the distinctive circatidal rhythmicity (period of about 12.4 h that coincides with the interval between the peaks of high and low tide) that intertidal species often display^52^.

Activity monitors can also be used to evaluate transient behaviours, as highlighted by the use of a DAM to detect activity peaks following blue light pulses in drosophila^8^. To provide users with this capability, Rtivity allows the visual inspection of transient behaviours and exports the represented data in a structured manner for further analysis (See “Integrated analysis of multiple experiments and software interoperability”). To illustrate this feature, we show in Fig. 3E the activity peak of a zebrafish stress response induced by a sudden light-to-dark transition, i.e. visual motor response^53^.

Thus, although the use of IR-based activity monitors and Rtivity is primarily advantageous over video-recordings for long-run experiments (days), its ability to use data from short-run experiments (minutes or hours) greatly expands its spectrum of applications. A key feature of Rtivity is that users can import data from multiple short-run experiments for a combined analysis, and then export the data in a friendly format for further analysis in specialized statistical software, as explained below.

### Integrated analysis of multiple experiments and software interoperability

**Figure 4:**
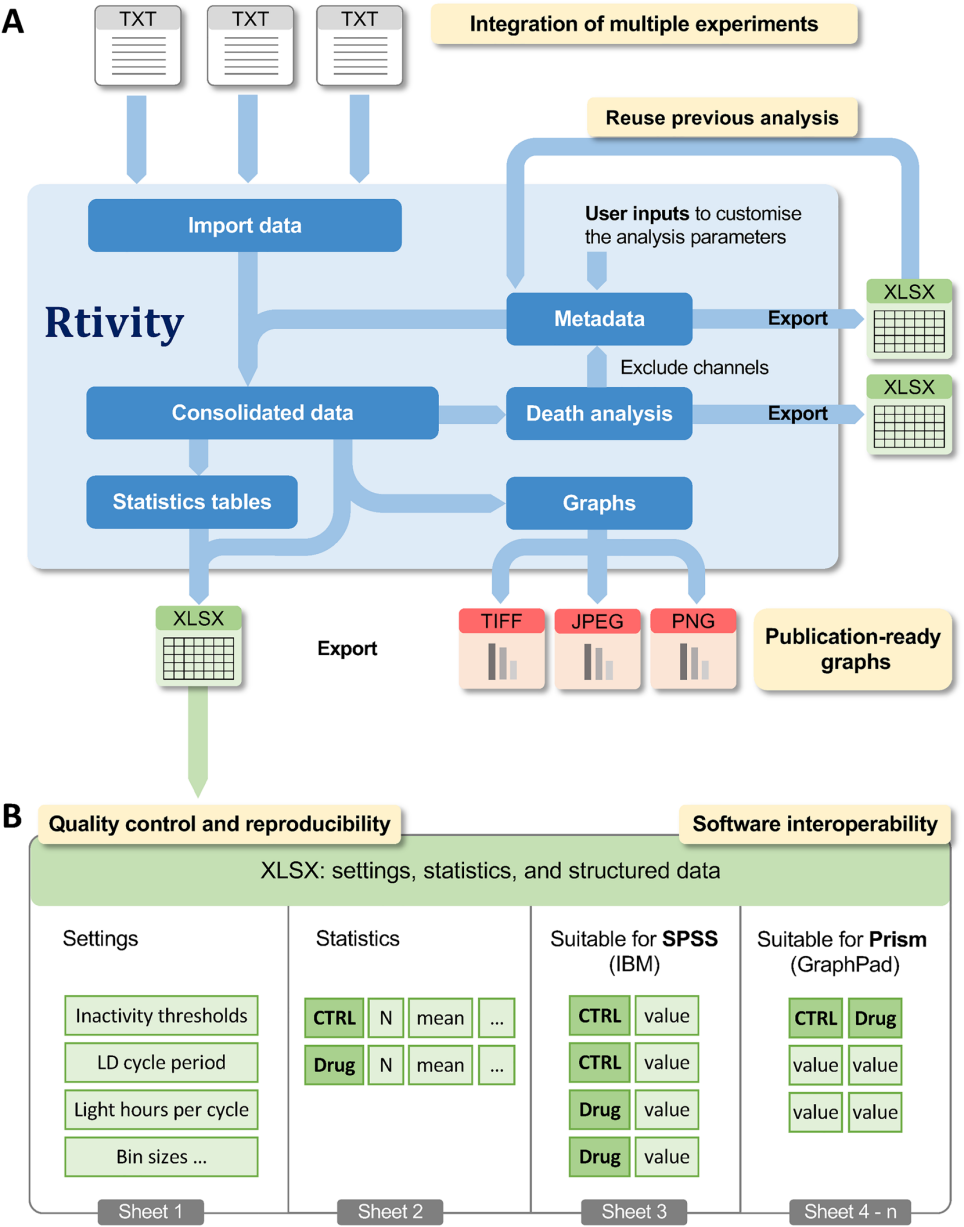
Software workflow and interoperability. (**A)** Rtivity can integrate multiple monitor files in the same analysis. From these files, users select conditions to analyse and input analysis parameters that become exportable metadata (XLSX), which can be reused to repeat/continue the analysis. Metadata contain: filename, start and finish dates, channels, labels (condition name), order (used to order the conditions in the graphs), and light-onset time (ZT0). Rtivity allows death analysis and exports (XLSX) the associated settings and results (survival status and time of death or last activity timepoint of all channels). Rtivity represents behavioural parameters in graphs and computes the statistics associated with each representation. Graphs can be customised for publication and exported in a user-selected format. (**B)** For quality control and reproducibility, Rtvity exports the settings associated with each analysis. For interoperability, Rtivity exports consolidated data, providing two common structures required by main statistical software (conditions in column vs. row). Figure was drawn by co-authors RS and JMAO using version 2.8.22 of the free image editor GIMP (https://www.gimp.org).

In research, robust results often emerge from the combined analysis of multiple independent experiments^54^. Rtivity allows the joint analysis of multiple monitor files (Fig. 4) and the combination of freely selected channels, from one or several files, into the same condition (Fig. 1C). Data from all imported files can be aligned by their individual light onset time (which may differ across files), thus allowing the integration of multiple experiments. Each analysis is defined by metadata (containing information identifying the files and selected conditions; Fig. 1C and Fig. 4), which users can save for further analysis or simply to keep track of the performed analysis.

Rtivity allows the export of all graphs, data and metadata, including user imputed settings (e.g. inactivity thresholds, bin sizes) to allow quality control and reproducibility (Fig. 4). Graphs are exported as TIFF, JPG or PNG files, while data and metadata are exported as XLSX files. The data represented in each graph can be exported in a structured manner to simplify the interoperability with commonly used statistical programs such as Prism (GraphPad) or SPSS (Statistical Package for the Social Sciences, IBM) (Fig. 4). The exported XLSX file includes the data from each individual channel in analysis, the settings imputed by the user, and the statistics associated with the graphical representation.

Rtivity’s ability to generate and (re)use metadata allows the simultaneous analyses of multiple experiments, and its structured data export capabilities greatly facilitates its direct use by third-party statistical software (interoperability) (Fig. 4).

### Software availability and user guide

Rtivity is available as an online application (without installation) and also as a desktop application, which has a simple installation process and is fully operational offline. In our Lab webpage: https://www.sites.google.com/view/mitoneuro/software) we provide a concise video demonstrating Rtivity data import, graphical representations, data export into xlsx files, and software interoperability with Prism (GraphPad; Fig. 4B). We also provide links to access the software, a user guide, and sample data for users to test in Rtivity. The online Rtivity version (https://ruisilva.shinyapps.io/rtivity/) is advantageous for quick exploratory analysis of small data sets (upload limit: 5 MB); and can be run from common web browsers without local installation. However, for improved speed and especially for larger datasets, we recommend the desktop application. This is because running Rtivity offline in the desktop application allows the upload of larger files and saves upload/download time, thus being significantly faster than the online version (shinyapps), which by third-party default disconnects after 15 min of idle time (no user inputs). The software scripts are available at GitHub (https://github.com/Rilva/Rtivity); for users who wish to run the software on Mac computers, it requires installation of R and RStudio, and the necessary libraries. The Windows version does not require additional installations, since the Rtivity_setup.exe file already includes a portable R with all the required libraries.

## Conclusion

In this work, we developed a software, called Rtivity, for the analysis of animal behaviour experiments. Rtivity uses data obtained from infrared activity monitors, or from other sources where activity is expressed in a counts per time format. Rtivity enables the analysis of different types of rhythms and behaviours displayed by a wide range of animal species, by allowing users to customise activity and sleep parameters, as well as light–dark cycles, in a user-friendly interface that does not require programming skills. Rtivity also expedites survival studies by automatically scoring and computing time of death. In Rtivity, users can integrate the analysis of multiple experiments, explore data visually with high graphical versatility, and export structured data for efficient software interoperability with common statistical software. Thus, Rtivity is suitable for a broad range of investigators, working in multidisciplinary research fields such as ecology, neurobiology, toxicology, and pharmacology.

### Ethical statement

Only young zebrafish larvae (maximum 7 days) were used in experiments. *N. lumbriciformis* was not used in experiments (data in Fig. 3D are a minor subset from a previous study, to demonstrate the software capability to use data from other sources and detect circatidal rhythmicity). All handling of animals, including zebrafish progenitors, followed the European Directive 2010/63/EU, and the Portuguese Law (Decreto Lei 113/2013), with procedures approved by the CIIMAR animal welfare body (ORBEA, Directive 2010/63/EU).
